# Descriptive analysis of unfavorable mental health opinions of candidates for bariatric surgery

**DOI:** 10.1192/j.eurpsy.2023.862

**Published:** 2023-07-19

**Authors:** R. C. Pereira, A. Delgado, F. M. Ferreira

**Affiliations:** 1 Hospital Magalhães Lemos; 2 Unidade Local de Saúde de Matosinhos, Porto, Portugal

## Abstract

**Introduction:**

In recent years, there has been an increase in the number of candidates for bariatric surgery and a decrease in psychiatric contraindications.

**Objectives:**

We aim to make an descriptive evaluation of unfavorable feedback concerning mental health of the candidates for bariatric surgery of the Local Health Unit of Matosinhos (Porto, Portugal).

**Methods:**

Descriptive analysis of unfavorable feedback of mental health of candidates for surgical treatment of obesity.

**Results:**

From March 2017 to August 2022, the Mental Health Service of the Local Health Unit of Matosinhos issued 347 pre-surgical feedback. In 63 cases the initial opinion issued was unfavorable: 11 cases due to a psychiatric contraindication (not meeting conditions for intervention) and 52 cases had a conditional opinion (requiring pre-surgical interventions in order to become eligible for the intervention). Regarding contraindications, these were due to alcohol use disorder (n=3), binge eating (n=3), intellectual development disorder (n=2), purgative behavior (n=1), psychotic disorder (n=1) and mood disturbance (n=1). In terms of conditional opinions, the issues mencioned were lack of motivation for surgery (n=22), psychopathology (n=20), doubts about informed consent (n=8) and need for multidisciplinary discussion/coordination (n= 7).

**Conclusions:**

There was an increase in eligibility of candidates for surgical treatment as most of the initial unfavorable opinions were conditional. This could be explained by the decline of complications associated with bariatric surgery, but also because psychiatric disorders are now being viewed as treatable. Notably people with eating disorders are now fit for surgery after a medical or psychotherapeutic intervention.

**Disclosure of Interest:**

None Declared

